# Supplementary material for: Over-Expression of Two Different Isoforms of Cattle TUSC5 Showed Opposite Effects on Adipogenesis
Source: Genes (Basel). 2022 Aug 14;13(8):1444. doi: 10.3390/genes13081444 (PMC9408160; doi:10.3390/genes13081444)
Supplement: Supplementary file 1 [file genes-13-01444-s001.zip › genes-1834162-supplementary.pdf]

Table S1 Primers used to amplify TUSC5A and TUSC5B

Primer name	Primer sequences
TUSC5-F	ccg*GAATTC*ATGTATGGCCAACCCCGGACAG
TUSC5-R	cgc*GGATCC***CATCATCACCATCACCAT**CGTTTCTTCGGAACTGTAAAG

Note: lower case letters are protective bases; italic letters are restriction site; bold letters codes for 6His tag.

Table S2 Primers for qPCR analysis

Gene symbol	Primer Sequence（5'-3'）	PCR product length（ bp）
PPARG	TTGATTTCTCCAGCATTTCT<br>TGTTGTAGAGCTGGGTCTTT	205
FASN	CTGGCATTCGTGATGGAGTC<br>TGTTTCCCCTGAGCCATGTA	204
FABP4	CGACAGGAAGGTGAAGAGCA<br>ATTCCACCACCAGCTTGTCA	203
LPL	TCGCCTTTCTCCTGATGACG<br>GCAATCACACGGATGGCTTC	171
ACC1	CAAGTGCTCAAGTTTGGCGC<br>CAAGAACCACCCCGAAGCTC	221
GPDH	TGGCATATGTGGAAGCAGCA<br>TCCTCTGGTGACGGCATCTA	167
SCD1	CCCTCCGGAAATGAACGAGA<br>CAGAGCGCTGGTCATGTAGT	261
GLUT4	AATGTCCTTGCTCCAGCTCC<br>CACCGAGACCAACGTGAAGA	208
ACTB	ATGATATTGCTGCGCTCGTGG<br>TCCATGTCGTCCCAGTTGGT	241

Table S3 Statistics of apoptosis detected by flow cytometry

Group	Cellular debris (mean±sd)	Early apoptosis (mean±sd)	Advanced apoptosis (mean±sd)	Positive cell ratio (mean±sd)
TUSC5A	2.27±0.88%	1.93±0.68%[bc]	1.52±0.42%[bc]	94.3±1.93%
TUSC5B	3.79±0.32%	5.26±1.02%[a]	3.71±0.43%[a]	87.2±1.00%
Control	0.6±0.21%	1.77±0.06%[c]	0.88±0.19%[c]	96.7±0.45%

Table S4 Positive rate of TUSC5A-C3H10, TUSC5B-C3H10 and Control-C3H10

Group	positive rate (mean±sd)
TUSC5A-C3H10	98.1±0.15%
TUSC5B-C3H10	98.9±0.06%

Control-C3H10	99.9±0.03%

Table S5 Pathway enrichment for DEGs up-regulated in TUSC5A-C3H10 comparing to Control-C3H10

Pathway	Genes	FDR
PPAR signaling pathway	FABP4, FABP5, ACSL1, EHHADH, ADIPOQ, NR1H3, AQP7, LPL, ACADM, SCD1, PCK1, CPT1B	1.10E-08
Metabolic pathways	PFKFB3, ACSM5, GPT, LTC4S, ACACB, HACD2, AGPAT2, HK2, GYS2, LDHB, ALDH3B2, DBT, GPAT3, SCD1, ACADM, PCK1, PRODH, IDH3A, COX8B, DGAT2, ACSL1, ELOVL3, CMBL, CRLS1, CYP51, ACER2, HADHB, ALDH6A1, DHRS9, EHHADH, CYCS, KYAT1, CYP2E1, LPIN1, RDH1, PNPLA2	1.10E-07
Fatty acid metabolism	HADHB, ACSL1, EHHADH, ELOVL3, ACADM, SCD1, HACD2, CPT1B	2.37E-05
AMPK signaling pathway	GYS2, PFKFB3, ADIPOQ, SLC2A4, SCD1, PCK1, ACACB, ADIPOR2, CPT1B	2.19E-04
Adipocytokine signaling pathway	ACSL1, ADIPOQ, SLC2A4, PCK1, ACACB, ADIPOR2, CPT1B	5.36E-04
Propanoate metabolism	LDHB, ALDH6A1, EHHADH, DBT, ACACB	0.002167
Alcoholic liver disease	ADIPOQ, CYP2E1, ACADM, SCD1, LPIN1, ACACB, ADIPOR2, CPT1B	0.002167
Glycerolipid metabolism	DGAT2, LPL, GPAT3, LPIN1, AGPAT2, PNPLA2	0.002167
Insulin resistance	GYS2, NR1H3, SLC2A4, PCK1, ACACB, CPT1B, AGT	0.003494
Fatty acid degradation	HADHB, ACSL1, EHHADH, ACADM, CPT1B	0.010306
Valine, leucine and isoleucine degradation	HADHB, ALDH6A1, EHHADH, DBT, ACADM	0.013269

Table S6 Pathway enrichment for DEGs down-regulated in TUSC5B-C3H10 comparing to Control-C3H10

Pathway	Genes	FDR
Metabolic pathways	GALNT12, ACSM3, GALNT15, ACSM5, COX6A2, CNDP2, NSDHL, CYP2C70, MLYCD, MMUT, MCCC2, DGAT1, ACSL1, TALDO1, CMBL, DPM1, ALDH3A1, PHOSPHO1, GAPDHS, SMS, CYP2E1, ATP6V0D2, PFKFB1, MAOA, PDE1B, NDUFB11, NPR1, MGST3, MGST1, AKR1C18, COX7A1, MTM1, HSD11B1, ALDH3B3, ALDH3B2, EBP, GM3776, AMDHD2, CHST10, PLCG2, RDH16, PRODH, GSTM4, MGAM, PDHA1, EPHX2, IDH1, CYP2J9, DOLK, ACER2, ALDH6A1, VNN1, VNN3, CYCS, KYAT1, ACO2, ALAS2, ST6GALNAC2, MTMR14, SUV39H1, POMT1, GPT, PYGL, AKR1B3, AKR1B7, GYS2, NAT8F3, GYG, CYP2B10, NAT8F1, SCD3, SCD1, DLAT, NAT8F7, ACADS, IDH3A, HGSNAT, PLA2G12A, COX8B, CYP4A10, PLA2G4B, BCKDHB, ELOVL3, GPX8, CTPS2, SIRT5, POMGNT2, RENBP, CAR5B, HADHB, CYP2J12, CYP26C1, EHHADH, G6PDX, ADSSL1, ACSBG1, ACOX3, NAT8L, RDH1, PLCB2, B4GALT7, ASPA, MGLL, BCAT2, PRPS2, AHCY, PCX, HSD17B4, LTC4S, ADCY8, HSD17B10, CYP7A1, AGPAT2, DDO, CBS, HSD17B1, MAN1C1, UGT1A2, UGT1A8, XDH, CAR2, HSD3B7, PLA2G2E, MDH1, IDH3G, GCK, GSTA2, GSTA1, PNPLA3, LPIN3, PNPLA2	1.25E-08
PPAR signaling pathway	ACSL1, CYP4A10, ADIPOQ, APOC3, NR1H3, AQP7, LPL, CYP7A1, CPT1B, FABP4, CPT2, FABP5, EHHADH, PLIN4, SCD3, OLR1, PLIN2, ACSBG1, PLIN1, ACOX3, SCD1, PPARA, PLIN5	1.25E-08
Drug metabolism - cytochrome P450	GSTM4, MAOA, MGST3, MGST1, FMO2, FMO3, ALDH3B3, ALDH3B2, ALDH3A1, GM3776, GSTA2, GSTA1, CYP2E1, UGT1A2, UGT1A8	4.37E-04
Peroxisome	PEX16, ACSL1, PEX11A, IDH1, EPHX2, HSD17B4, DDO, PEX26, GNPAT, EHHADH, PEX6, PXMP2, ACOX3, MLYCD, XDH, CRAT	7.33E-04
beta-Alanine metabolism	ALDH3B3, ALDH3A1, ALDH3B2, ALDH6A1, EHHADH, MLYCD, ACOX3, ACADS, CNDP2	0.005095
Glutathione metabolism	GSTM4, MGST3, IDH1, MGST1, GPX8, NAT8F3, GM3776, GSTA2, NAT8F1, GSTA1, G6PDX, SMS, NAT8F7	0.005095
Fatty acid metabolism	HADHB, CPT2, ACSL1, EHHADH, ELOVL3, HSD17B4, SCD3, ACSBG1, SCD1, ACOX3, ACADS, CPT1B	0.005095

Metabolism of xenobiotics by cytochrome P450	GSTM4, MGST3, MGST1, ALDH3B3, HSD11B1, ALDH3B2, ALDH3A1, GM3776, GSTA2, GSTA1, CYP2E1, UGT1A2, UGT1A8	0.005095
Carbon metabolism	PRPS2, PDHA1, PCX, MDH1, IDH3G, IDH1, TALDO1, GPT, GCK, ALDH6A1, G6PDX, ACO2, DLAT, ACOX3, MMUT, ACADS, IDH3A	0.00553
Citrate cycle (TCA cycle)	PDHA1, PCX, MDH1, IDH3G, IDH1, ACO2, DLAT, IDH3A	0.017288
Regulation of lipolysis in adipocytes	LIPE, FABP4, NPR1, ADORA1, AQP7, PLIN1, ADCY8, MGLL, TSHR, PNPLA2	0.029526
2-Oxocarboxylic acid metabolism	IDH3G, IDH1, GPT, ACO2, BCAT2, IDH3A	0.044523
Hepatitis C	SCARB1, STAT1, MX2, STAT2, OAS1A, NR1H3, TYK2, IFIT1BL1, IFIT1, IFIT1BL2, OAS1G, OAS2, OAS3, IRF7, CYCS, IKBKG, PPARA, IRF9	0.044523
Arachidonic acid metabolism	PLA2G12A, CYP2J12, PLA2G2E, CYP2B10, PLA2G4B, EPHX2, CYP4A10, CYP2J9, GPX8, CYP2E1, CYP2C70, LTC4S	0.047182
